# Short-term synaptic plasticity in the nociceptive thalamic-anterior cingulate pathway

**DOI:** 10.1186/1744-8069-5-51

**Published:** 2009-09-04

**Authors:** Bai-Chuang Shyu, Brent A Vogt

**Affiliations:** 1 Institute of Biomedical Sciences, Academia Sinica, Taipei, 11529, Taiwan, Republic of China; 2 Cingulum NeuroSciences Institute and SUNY Upstate Medical University, Syracuse, NY 13210, USA

## Abstract

**Background:**

Although the mechanisms of short- and long-term potentiation of nociceptive-evoked responses are well known in the spinal cord, including central sensitization, there has been a growing body of information on such events in the cerebral cortex. In view of the importance of anterior cingulate cortex (ACC) in chronic pain conditions, this review considers neuronal plasticities in the thalamocingulate pathway that may be the earliest changes associated with such syndromes.

**Results:**

A single nociceptive electrical stimulus to the sciatic nerve induced a prominent sink current in the layer II/III of the ACC *in vivo*, while high frequency stimulation potentiated the response of this current. Paired-pulse facilitation by electrical stimulation of midline, mediodorsal and intralaminar thalamic nuclei (MITN) suggesting that the MITN projection to ACC mediates the nociceptive short-term plasticity. The short-term synaptic plasticities were evaluated for different inputs *in vitro *where the medial thalamic and contralateral corpus callosum afferents were compared. Stimulation of the mediodorsal afferent evoked a stronger short-term synaptic plasticity and effectively transferred the bursting thalamic activity to cingulate cortex that was not true for contralateral stimulation. This short-term enhancement of synaptic transmission was mediated by polysynaptic pathways and NMDA receptors. Layer II/III neurons of the ACC express a short-term plasticity that involves glutamate and presynaptic calcium influx and is an important mechanism of the short-term plasticity.

**Conclusion:**

The potentiation of ACC neuronal activity induced by thalamic bursting suggest that short-term synaptic plasticities enable the processing of nociceptive information from the medial thalamus and this temporal response variability is particularly important in pain because temporal maintenance of the response supports cortical integration and memory formation related to noxious events. Moreover, these modifications of cingulate synapses appear to regulate afferent signals that may be important to the transition from acute to chronic pain conditions associated with persistent peripheral noxious stimulation. Enhanced and maintained nociceptive activities in cingulate cortex, therefore, can become adverse and it will be important to learn how to regulate such changes in thalamic firing patterns that transmit nociceptive information to ACC in early stages of chronic pain.

## Introduction

The cingulate cortex is one of the most frequently activated regions in human pain research [1,2]. The thalamus is also frequently activated and its responses are correlated with the nociceptive responses in the cingulate cortex [3,4]. The cingulate response, however, may not be stable over time. A human imaging study has shown that the cingulate noxious activation habituates over time, while innocuous responses are not altered [5]. Response variability over time is particularly important in pain processing as the temporal maintenance or habituation of the response supports cortical integration and memory formation. Thus, the temporal characteristics of synaptic plasticity from peripheral to cortical targets are pivotal to understanding pain processing, anticipation of future pain events and developing avoidance behaviors.

Of equal importance is the fact that anterior cingulate cortex (ACC) has been implicated in a number of human chronic pain syndromes and three studies activated pregenaul ACC. Kern et al. [6] stimulated the esophagus with acid to induce heartburn in gastroesophageal reflux disease patients and Naliboff et al. [7] and Mayer et al. [8] employed noxious rectal distension in patients with irritable bowel syndrome. Frequent migraine and tension-type migraines are associated with reduced grey matter in ACC [9,10]. Thus, short- and long-term plasticities may subserve the initiation of chronic pain states in ACC and we consider the short-term plasticity in this review. For reviews on long-term plasticity changes in the ACC, see Zhou's articles [11-14].

Neurons communicate with each other by transmission through chemical synapses and the dynamic course of synaptic transmission is regulated by a variety of short-lasting processes. The sum of pre- and post-synaptic responses evoked by stimulation of afferent axons is often termed synaptic "strength" and during dynamic short-term processes it varies in a systematic manner and is dependent on the precise onset and duration of activation, i.e., tens of milliseconds to several minutes. Short-term plasticities (STP) have been described in several forms, such as paired-pulse facilitation (PPF), augmentation, post-tetanic potentiation and synaptic depression which are each distinguished by their decay kinetics [15].

PPF is the synaptic enhancement of the second response in which the post-synaptic potential is increased up to several times the amplitude of the first potential. The enhancement of synaptic potentials can develop and decline with a time course of about 100 ms. When the response lasts for 5-10 s, it is termed synaptic augmentation and is distinguished from post-tetanic potentiation which lasts from 30 s to several minutes. Furthermore, post-tetanic potentiation is an augmentation of synaptic transmission following a train of repetitive stimuli. During the stimulation each synaptic potential increases the synaptic strength by 1-15% and the summed effect of sequential pulses can reach to a many-fold enhancement.

Action potential discharges are often activated at high frequencies or in a bursting mode and it is the pattern of dynamic discharges that change with altered synaptic strength. Thus, the properties of STP that are specific to the activated synapses may determine the spiking pattern of a presynaptic cell that ultimately influences the firing of its post-synaptic neurons. Many mechanisms can lead to activity-dependent alterations in synaptic strength during neuronal high-frequency discharges. A short-term depression may result from a reduction in neurotransmitter release either by reducing the probability of release or by depleting the readily releasable pool of vesicles. A short-term facilitation may occur by repeated activations that increase the probability of neurotransmitter release, either by saturating a local calcium buffer or by increasing calcium concentration in the presynaptic terminal [16,17]. The post-synaptic mechanism may also involve regulation of the properties of the STP [18]. For instance, desensitization of postsynaptic receptors by neurotransmitters can reduce synaptic responses during repeated activation; however, the presynaptic and postsynaptic mechanisms may only partially determine the properties of the STP. The variety of plasticities exhibited by different synapses reflects the many of functions that synapses serve in extracting features of presynaptic activity. Multiple mechanisms are present at most synapses that interact and lead to complex responses during patterns of synaptic activation.

Short-term synaptic plasticity appears to be a widespread in the nervous system and STP is a dominant mechanism underlying the plasticity of sensory responses in mammalian cerebral cortex. The functional relevance of the short-term synaptic depression and facilitation has been linked to habituation and sensitization, respectively. Recently, studies have extended its role in this simple form of learning and it has been suggested that STP involves signal filtering that is used in information processing. Specifically, the differential activation and integration of short-term synaptic depression and facilitation might enhance and sustain temporal filtering [18].

At the simple reflex level in the spinal cord, the STP of inhibitory interneurons provides an intrinsic mechanism for the dynamic regulation of a reflex. At higher levels of the nervous system, such as in the cerebral cortex, STP provides a dynamic mechanism for signal processing where it may serve as a general mechanism for the short-term amplification of signals. Some evidence suggests that short-term facilitation participates in temporal coding within neuronal circuits [19]. Furthermore, it has been shown that short-term facilitation coupled with slow inhibitory conductances might act to transform temporal codes to spatial codes within a cortical circuit [20]. New findings also suggest that short-term facilitation and synaptic depression can interact in the neocortex in complex functions such as visual contrast adaptation and enhanced sensitivity to dynamically changing cortical inputs [21]. Thus, when considering diverse systems, STP appears to provide a highly flexible and adaptive mechanism that might contribute significantly to temporal information processing ranging from single synapses to complex neural circuits involving many classes of interneurons. Studies in STP in the thalamocortical pathways have furthered our understanding of the dynamic cortical processes activated by specific inputs. Indeed, STP in the thalamocortical circuit may play an important role in pain processing.

### Relationship of STP to Nociception at Each Level of the Nervous System

All levels of pain processing in the CNS are associated with STP. Temporal enhancement of nociceptive signals throughout the nervous system provide a mechanism of amplifying one or a few signals above baseline activity over time and the thalamus and cortex have time to assess the signals in context; i.e., in the context of other visual, auditory or somatic sensory events. In the longer term, this information can be used to predict painful outcomes, establish new memories, and modify nocifensive reflexes to particular contexts.

#### Nociceptors

In the peripheral nervous system, nociceptors are distinct from innocuous sensory receptors in that they have a high threshold of activation. The responses of mechanoheat-sensitive nociceptor axons increase monotonically with heat stimuli ranging from 41-49°C and correlates with the pain threshold in humans [22]. The peripheral neural mechanisms of nociception reflect only one aspect of pain sensibility, since there is a dynamic plasticity that relates stimulus intensity and sensation. For instance, the response of nociceptors is strongly influenced by the past history of stimulus sequence. Both fatigue and sensitization of nociceptors following repetitive heat or mechanical stimuli have been observed. [22-24]. Furthermore, tissue damage can result in a cascade of events that leads to enhanced pain in response to noxious stimuli which is termed hyperalgesia. This type of primary hyperalgesia exemplifies the functional plasticity of the nervous system. Substantial evidence supports the view that the primary hyperalgesia to heat and mechanical stimuli that develops at the site of a injured area is mediated by the sensitization of nociceptors [25,26]. Sensitization is characterized by an enhanced nociceptive response to supra-threshold stimuli in addition to a decrease in threshold and ongoing spontaneous activity.

#### Spinal cord

Trains of afferent discharges following repeated noxious inputs from nociceptors can evoke a period of facilitated transmission in dorsal horn neurons in the spinal cord. Windup is a form of STP characterized by a progressive increase in action potential output from dorsal horn neurons during a train of repetitive, C-fiber stimuli [27,28]. It appears that this form of STP in the dorsal horn plays an important role in post-injury pain hypersensitivity and in the initiation of the persistent pain. Pain signals in this context serve as a warning signal for the organism and the nociceptive system may increase its sensitivity following exposure to repetitive noxious stimuli that result in sensitization. This sensitization enhances escape responses and, with a reduced threshold, protects the organism from further injury.

Nociceptive information transmitted from the dorsal horn to the forebrain are encoded in lamina I and lamina V neurons with nociceptive-specific (NS) and wide dynamic range (WDR) properties. Spinal nociceptive neurons project to the reticular formation, midbrain periaqueductal grey, parabrachial nucleus, dorsal column nuclei and thalamus [29-33]. Neurons that form the spinothalamic pathway show graded responses to innocuous and noxious mechanical stimuli, noxious heat and cold and noxious muscle or visceral stimuli. Their responses usually increase with strong noxious stimulation followed by sensitization to innocuous stimuli in a manner that resembles the hyperalgesia and allodynia experienced by humans following such stimuli [34]. On the other hand, the lamina I NS and polymodal NS cells can readily be sensitized to innocuous mechanical and cold thermal stimuli by repeated noxious stimulation [35]. A unique study in humans used repetitive electrical stimulation of anterolateral fibres of the spinal cord and showed a linear relationship between stimulation frequency and the subject's pain [36]. This relationship ranged from 5-25 Hz with 100% of patients reporting pain at 25 Hz and 0% at 5 Hz. The threshold, frequency and refractory period data obtained are similar to those for WDR cells in the ventral half of the dorsal horn in the monkey and suggest that activation of these cells is a sufficient condition to produce human pain. Since the pain was blocked by anterolateral cordotomy that removes spinothalamic afferents in these patients, it is likely that such information is transmitted to the thalamus.

#### Thalamus

The thalamus is the essential relay of nociceptive inputs that are transmitted from the spinal cord to cortex. The likely thalamic source of such information to anterior cingulate cortex (ACC) is from the midline, mediodorsal and intralaminar thalamic nuclei (MITN) [37,38]. As noted earlier, high frequency discharges or bursting alter synaptic strength, i.e., STP that are specific to the activated synapses determine the spiking pattern of postsynaptic neurons. The bursting discharge pattern is well known in the thalamus and it has been reported that bursting discharges similar to the low-threshold calcium spike-mediated bursting activity in animal studies exists in the thalamus of chronic pain patients [39,40].

There is also evidence for plasticity in the thalamocortical system in pain patients with deafferentation as a result of amputation or spinal cord injury. This implies that neuronal activity in the thalamus gives rise to sensations perceived as originating from the missing part of the original limb. Furthermore, stimulation in such regions frequently elicits pain sensations arising from the deafferented body region [41].

The role of the STP in nociceptive signalling in the thalamocingulate pathway is still not clear. Attempts have been made to link the short-term facilitation to synapses in this pathway and their role in temporal signal integration. The cellular mechanisms underlying these dynamic responses involve pre- and post-synaptic and circuit properties [42] and the short-term facilitation could act to enhance the ability of a neuronal circuit to sustain persistent activity evoked by a transient stimulus. Also, enhancement of synaptic transmission in short periods of time could temporarily increase the level of recurrent excitation throughout a cortical network. Such synaptic enhancement is a dynamic mechanism for temporarily enhancing the efficacy of recurrent synapses. Thus, the STP may serve to synchronize, amplify and/or filter neural activity in cortex depending on behavioral demands, and thus to adapt this pathway to its specific nociceptive function.

### Thalamic Nociceptive Transmission to Anterior Cingulate Cortex

**Figure 1:**
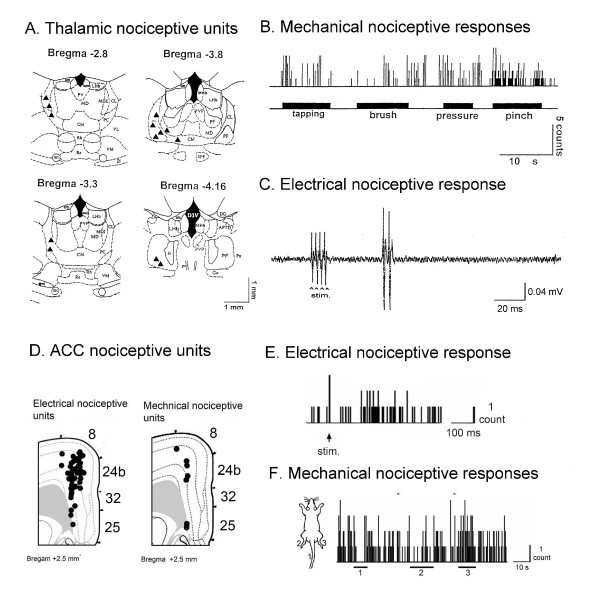
**Thalamic and ACC nociceptive unit responses and distribution**. **A**. Thalamic distributions of electrical and mechanical evoked unit responses. **B**. Thalamic unit responses evoked by noxious mechanical stimuli. **C**. Thalamic unit response evoked by electrical stimuli. **D**. Cortical numbers for each ACC area and layer distributions of the electrical and mechanical evoked unit responses in the ACC. **E**. Unit activities evoked by noxious mechanical stimuli. The horizontal black bars indicate the time periods during which the following stimuli were applied: 1. tail, 2. left hind paw, and 3. right hind paw. **F**. Post-stimulus histogram of electrically evoked unit activities in the ACC (Modified from 42 and 44).

Nociceptive responses are transmitted to cingulate cortex from the MITN. Thus, expression of the product of the immediate early c-fos gene is a marker of metabolic activity and c-Fos expression in the MITN is increased in rats subjected to noxious colorectal distension or electrical stimulation of hind limb C-fibers [43]. Neurons in the MITN have nocireceptive response properties [44,45] and these are reflected in neuron responses of ACC. Figure 1 shows neuronal responses in the mediodorsal (MD) and centrolateral (CL) thalamic nuclei during nociceptive stimulation [46]. Most of the MITN neurons (78%) responded to both peripheral innocuous and noxious stimuli and were WDR neurons. The remainder of the units (22%) showed NS responses. The receptive fields of MITN nociceptive neurons are widely distributed on the body surface and covered the two lower extremities and/or the entire body. In some instances, electrical stimulation applied to the center of the receptive fields and the mean latency of responses evoked in MITN neurons was 41.98 ± 1.4 ms (Mean ± S.E.M.).

Several lines of evidence indicate that the MITN provide the primary source of nociceptive information to the ACC.

**First**, the nociceptive response in rabbit ACC neurons occurred within 200 ms which may preclude prior processing via other cortical areas. Also, knife-cut lesions lateral or posterior to ACC that remove most cortical input to ACC do not alter the percentage of units in area 24 driven by noxious stimuli [47].

**Second**, the MITN has similar nociceptive response properties with cingulate neurons, which suggests a functional linkage. Nociceptive ACC neurons have a broad somatotopic organization; i.e., stimulation of large parts of the body can activate a single ACC neuron. They also respond to noxious mechanical or heat stimulation on both sides of the body and can be polymodal in responding to both such stimuli. In other words, single ACC neurons do not "know" where on the body the stimulus is occurring and often do not "know" what type of stimulus is producing the pain. Electrically-evoked cutaneous nociceptive responses in the ACC are depicted in Figure 1E[48]. Each cingulate area is shown (areas 24b, 32, 25) along with adjacent motor area 8 (Fig. 1D). Fifty-five percent of total recorded neurons were excited or inhibited either by noxious electrical or mechanical stimulation. Among the electrical stimulation-responsive neurons, 88% had excitatory responses and 12% had inhibitory responses, while the percentage of neurons responsive to noxious mechanical stimuli was either excitatory (78%) or inhibitory (22%). Finally, most responsive neurons were in layers V (58%) and III (30%). A typical example of an ACC unit response to noxious mechanical stimulation is shown in Figure 1F where one unit had excitatory responses to left hind paw (3) pinch, but inhibitory responses to both tail (1) and right hind paw (2) pinches.

**Third**, thalamic lidocaine injections block ACC nociceptive activity [47].

**Fourth**, electrolytic lesion of MITN activity abolishes nociceptive responses in ACC [49].

**Figure 2:**
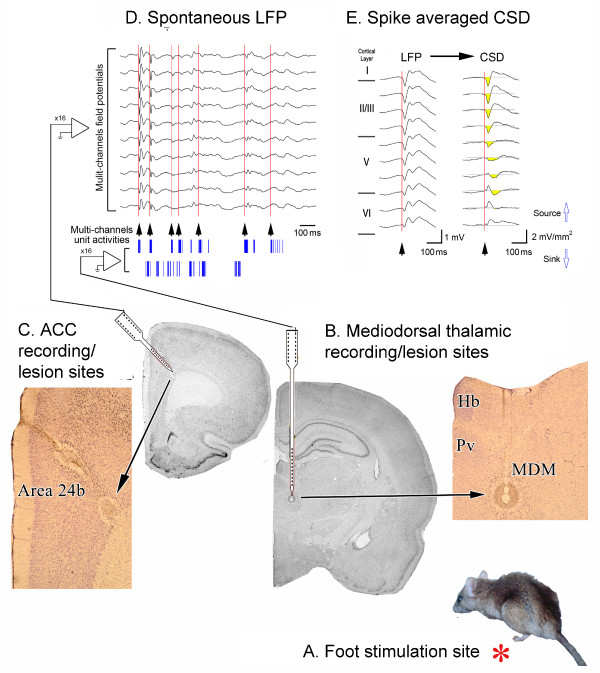
**Thalamocingulate responses during formalin injection to the hind paw (A, red asterisk)**. Mulichannel unit activities and local field potentials were recorded from the MITN (**B**) and ACC (**C**) respectively. **D**. Aligned multichannel thalamic unit activities and ACC spontaneous local field potentials showing that bursting activity and local field potential in both structures is correlated. **E**. The initial time of bursting (arrow heads aligned at the red lines) of MITN unit activities were aligned. Current source density profile across the cingulate cortical layer was calculated from the local field potentials. Abbreviations: MDM, mediodorsal thalamic nucleus, Pv, paraventricular thalamic nucleus, Hb, habenular nucleus.

**Fifth**, multichannel recordings from all layers of ACC and thalamus during tonic noxious formalin hind paw injections confirm the nociceptive thalamocingulate link. Lee et al. [50] showed there is a high correlation between MITN nociceptive-related neuron activity and local field potentials in the ACC. Figure 2A, B, and 2C show the formalin-injection model and joint recording paradigm. In addition to the high correlation of spike outputs from both structures, bursting discharges were induced in thalamic neurons (Fig. 2D) and could be used to align spontaneous local field potentials (Fig. 2D.) and averaged current-source-density (CSD) derived from the local field potentials (Fig. 2E.). The bursting discharges were used to align field-potentials across all layers of ACC and this demonstrated the laminar profile of local field potentials and the synaptic activation of ACC evoked activity by MITN afferents.

### A Remark on Current Source Density Analysis

One of the problems in interpreting the source localization of field potentials is that the voltage traces reflect both local and volume conducted activities. Thus, the values of the voltage activity pattern and those of the frequency power spectrum do not reflect local activities in particular cortical layers when the voltage is considered alone. When studying the laminar distribution of spontaneous electrical rhythms with a single electrode, it is difficult to predict the location of the current source of the synaptic input because of the variability of the rhythms in amplitude, frequency, and location during successive events. The ACC is dominated by parallel-aligned pyramidal cells whose apical dendrites extend across many cortical layers. Synchronous excitation of ensembles of pyramidal cells results in a major current flow perpendicular to the cortical layers. Under the assumptions of homogeneous cortical activity and constant extracellular electrical conductivity, CSD of the current flow can be estimated from the second spatial derivative of the recorded field potentials in the axis parallel to the cortical layers [51]. Thus the procedure employed to obtain CSD data is to record the field potential at equidistant, linearly positioned electrode contacts using multi-channel recording electrodes that vertically penetrate the cortical layers. This method can alleviate the problem of source localization and derive these sink current calculations from the CSD traces which reflect the actual local current flow. This is an important consideration, since the essence of the present study relates electrical patterns to laminar position.

### Short-term Plasticity in ACC

As a general rule, STP at cortical synapses strongly influences network activity [52-54]. Unlike the transient nature of the sensory responses in primary visual and somatosensory cortices, neurons in association cortices, including cingulate cortex, can exhibit persistent activity that outlasts the initial stimulus considerably [55-57]. Such activity may reflect activation of recurrent excitatory circuits or intrinsic synaptic plasticities. The major neurotransmitter involved in excitatory synaptic transmission in the ACC is glutamate and thalamic inputs are the primary source of glutamatergic signaling to cingulate neurons [49,58-60]. The blockade of thalamic-evoked intra-ACC sink currents with CNQX *in vivo *strongly indicates that AMPA/kainate glutamate receptors mediate the excitatory drive in thalamic inputs that are presynaptic to cingulate neurons [49]. *In vitro *whole cell patch-clamp recordings conducted in genetically modified mice show that postsynaptic kainate receptors contribute to fast synaptic transmission in ACC pyramidal neurons [42]. The functional activation of NMDA receptors in the ACC may require co-activation of glutamate- and glycine-binding sites. Whole-cell patch-clamp recording in ACC slices showed that endogenous D-serine may play a critical role in synaptic transmission by activating the glycine site of NMDA receptors in the ACC [61]. GABA is an important inhibitory neurotransmitter mediating the excitatory synaptic transmission in the ACC. Thalamocingulate terminals synapse on GABAergic interneurons in addition to principal neurons in the ACC [62]. This arrangement may enable GABAergic interneurons to inhibit cingulate principal neurons by feedforward inhibition [63]. On the other hand, it has also been shown that GluR5 containing kainite receptors also modulate GABAergic transmission in the ACC [43]. The presence of GABAergic terminals, both pre- and post-synaptic of thalamocingulate synapses, may enable disinhibition of interneurons after activation of thalamocingulate afferents [59,64,65].

Thus far, it appears that stimulation of any afferent to ACC induces STP and distinct forms of short-term synaptic plasticities in the cingulate neurons have been reported in studies of electrical stimulation of callosal inputs [66], the layer II/III junction [67], layer V [65], and the thalamocortical pathway [58,68]. Short-term depression and facilitation are similar to those described previously in other sensory cortical regions discussed above. In addition, synapses in cingulate cortex express augmentation; a longer lasting form of short-term synaptic enhancement. This consists of a 40-60% enhancement of synaptic transmission which lasts seconds to minutes and that can be induced by stimulus trains of moderate duration (15 stimuli) and frequency (50 Hz). The hypothesis guiding our studies of the thalamocingulate circuit is that the nociceptive MITN input generates STP in ACC and may be a precursor to longer term pain processing events.

### Peripheral Nociceptive and MITN Stimulation

**Figure 3:**
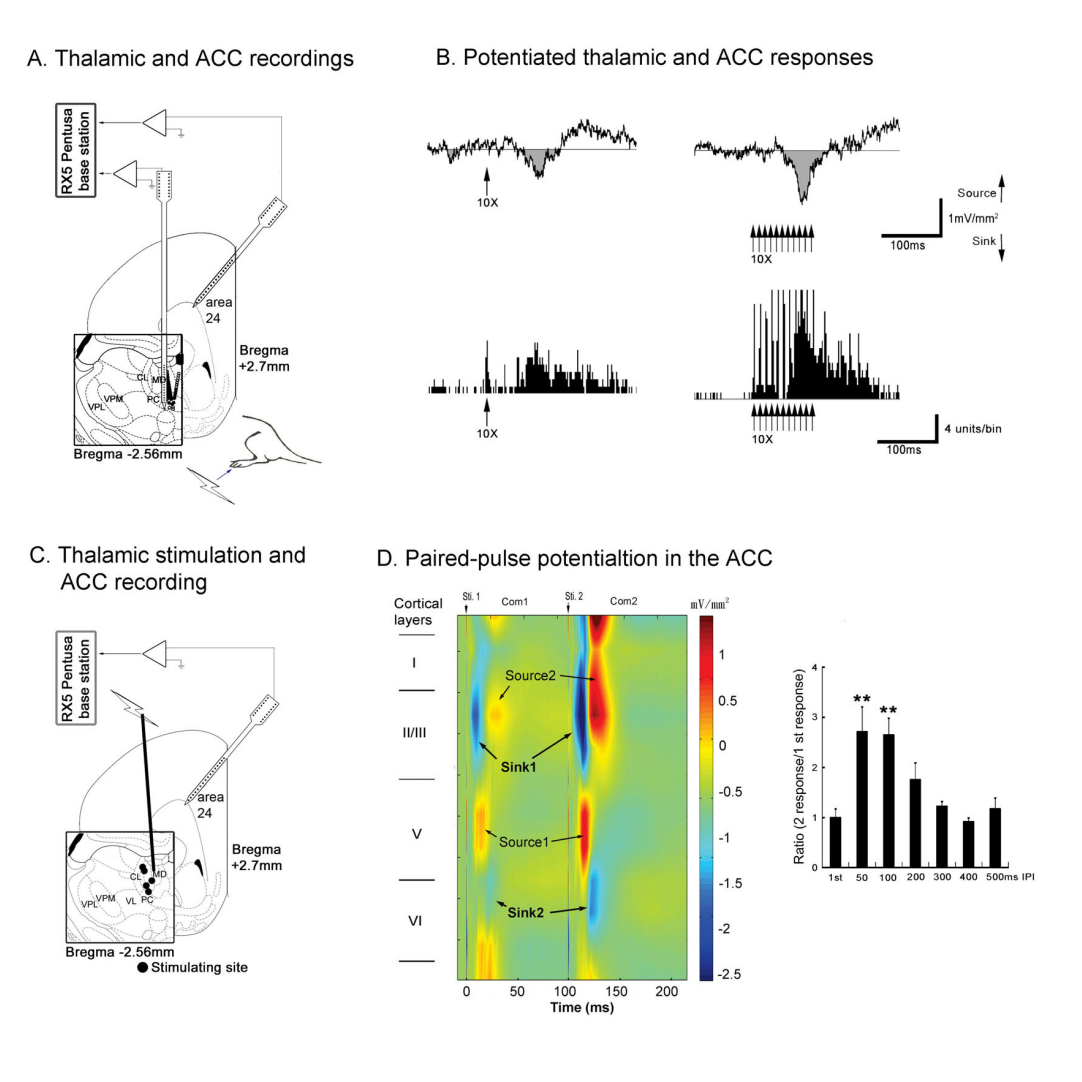
**Effects of high-frequency, paired-pulse stimulation on ACC responses *in vivio***. **A**. Diagram depicting the locations of the 2 multichannel probes used to simultaneously record activities from the MD thalamic nucleus and ACC. **B**. Example of enhanced ACC responses and MITN unit activities after high-frequency sciatic nerve stimulation (100 Hz, 11 pulses). **C**. Diagram of a multichannel probe used to record activities from the ACC and a tungsten electrode used to stimulate the MD nucleus. Black dots represent the stimulating sites. **D**. evoked CSD profile after direct stimulation of the MD nucleus. Paired-pulse facilitation was observed in ACC layer II/III CSD responses evoked by direct electrical stimulation of the MD nucleus when pulses were delayed 50 to 100 ms (*n *= 5; **P < 0.01). Abbreviations: CL, centrolateral thalamic nucleus; MD, mediodorsal thalamic nucleus; PC, paracentral thalamic nucleus; VL, ventrolateral thalamic nucleus; VPL, ventral posterolateral thalamic nucleus; VPM, ventral posteromedial thalamic nucleus (Modified from 45)

A single nociceptive electrical stimulus (10 mA) to the sciatic nerve induced a prominent sink current in the layer II/III of the ACC (Fig. 3B, left plate). High frequency stimulation of the nerve (11 pulses, 100 Hz) potentiated the evoked response of sink currents in layers II/III and V (Fig. 3B, right plate). There was a strong correlation (r = 0.91, p < 0.001) between MITN neuron activities and the integrated layer II/III sink currents [49].

One test to show the involvement of the MITN in transmitting this STP is to evaluate paired-pulse stimulation in these nuclei directly as shown in the bottom half of Figure 3. The first response to a paired-pulse thalamic stimulation evoked a sink current in layer II/III as shown in blue (sink 1, Fig. 3D, left plate). The complementary source current (source 1, yellow) appeared below in layer V. The second sink current (sink 2) was activated with a longer latency and situated in upper layer VI. The complementary source current (source 2) was in layer II/III. These sink currents were potentiated by the second pulse (dark blue, Fig. 3D, left panel) relative to the first responses. The potentiation was significant when the inter-pulse interval was in the 50-100 ms range (Fig. 3D, right plate). These two observations together suggest that the MITN projection to ACC mediates the nociceptive STP.

### Unique Features of MITN/ACC STP

**Figure 4:**
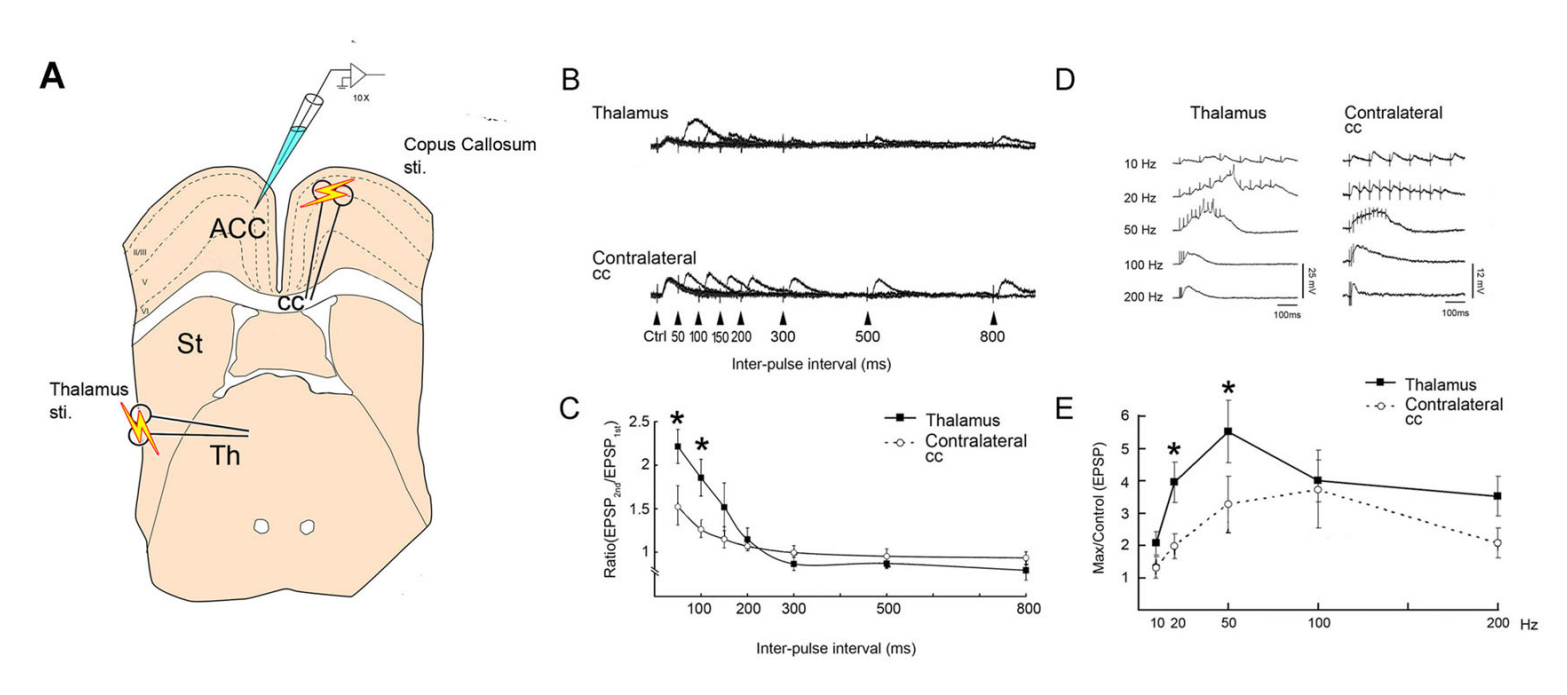
**PPF and tetanic potentiation of the ACC evoked by stimulation of the thalamus or the contralateral corpus callosum (CC)**. **A**. Schematic diagram showing stimulation and recording sites in an ACC slice model. **B**. Example traces of EPSPs evoked by different inputs at varying paired pulse intervals (50-800 ms). **C**. A greater paired-pulse potentiation ratio (second EPSP/first EPSP) at the 50- and 100-ms intervals was observed with stimulation in the thalamus than in the contralateral CC (*P < 0.01 vs stimulation in contralateral CC). Data are means ± SEM. **D**. Example traces of tetanic potentiation in the ACC evoked by stimulation of the thalamus, or contralateral CC. **E**. Potentiated responses (Max/Control EPSP) were greater with thalamic stimulation than with contralateral CC stimulation, at 20 and 50 Hz (*P < 0.01 vs contralateral CC stimulation). Data are means ± SEM (Modified from 46).

As we anticipate that STP has a role on information processing, it may provide a mechanism to distinguish the information from different inputs. To examine this hypothesis in MITN-ACC pathway *in vitro*, a slice preparation was developed with this pathway intact [50]. ACC electrophysiological studies *in vitro *have been carried out as shown in the Figure 4. This slice preparation has intact MITN-ACC path and corpus callosum (cc) stimulation can be used to compare STP induced from both sites (Fig. 4A).

We found that MITN-stimulation produces marked short-term facilitation in the ACC. Paired-pulses were delivered with varied (50, 100, 150, 300, 500, 800 ms) inter-pulse intervals (IPIs) at 80% of the intensity that induced maximum response activity (Fig. 4B). Maximal potentiation was obtained with a 50 ms IPI, and PPF with 50 ms and 100 ms IPIs was significantly greater when the stimulation was delivered in the thalamus rather than in the contralateral cc (Fig. 4C). Tetanic stimuli applied to the thalamus enhanced excitatory postsynaptic potentials (EPSPs) at all frequencies tested (10-200 Hz) and the maximal potentiation was obtained with 50 Hz stimulation (20 ms IPI) (Fig. 4D). The thalamus-evoked potentiated responses with 20 Hz and 50 Hz stimulation were significantly greater than that evoked by from the other site (Fig. 4E).

### Analysis of Thalamic Bursting Activities during Noxious Stimuli

**Figure 5:**
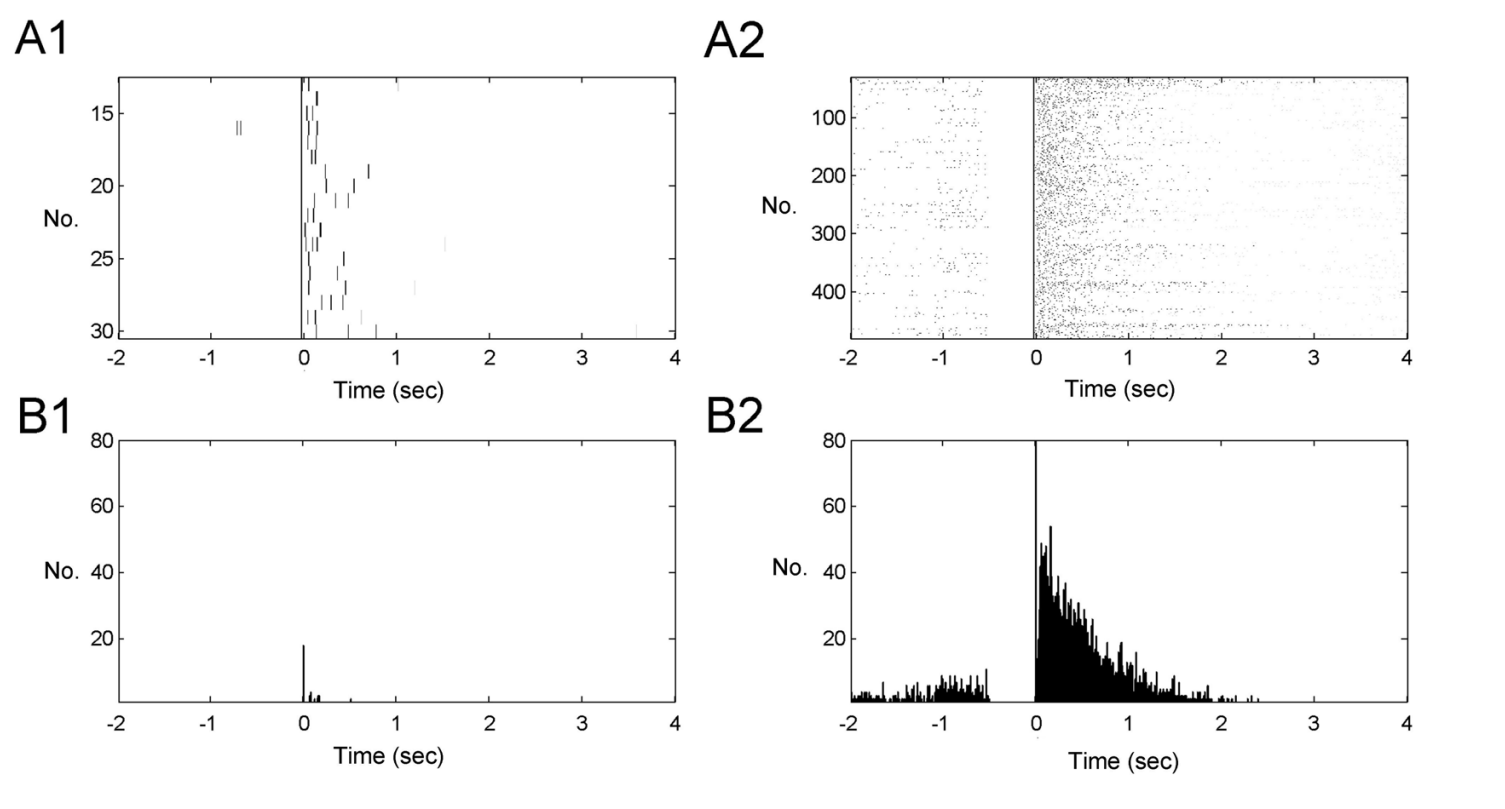
**Bursts analysis of MITN pattern in control and after formalin injection**. Burst unit activities were displayed as raster plot of under control (A1) and formalin injection (A2) condition. The spikes were sorted by its shape at first. Then, the spikes within one burst were separated from other spikes with a 500 ms pre-burst silent interval. All spike activities were shown as black dots in raster plot. The burst-trigged histogram of spike activities in control (B1) and formalin injected (B2) condition were summated from raster plot with a 10 ms bin width.

The tetanic stimuli used above were pulses delivered with a regular pulse sequence but action potential discharges are often activated in irregular bursting mode in physiological conditions. Thus, the properties of STP that are specific to the activated synapses may be tested in a spiking pattern that mimics the firing condition during nociceptive stimulation. We recorded unit activities in MITN which received inputs from hind paw of anesthetized rats. The burst pattern of thalamic units was analyzed under pre- and post- formalin injected conditions. Following the formalin injection in the hind paw, the increased firing rate of unit activity was regarded as nociceptive. Unit activities were collected 30 min before and one hour after the formalin injection. The increased unit activity can be expressed as increasing firing rate in the burst and the burst sequences were defined with at least 500 ms pre-burst silent period and at least 3 spikes within 300 ms post-burst period. All burst related unit activities were displayed in raster plot (Fig. 5A). Burst sequences (shown as black dot) were aligned by first spike of bursts which separate the pre- and post-burst spikes. There is a significant increase in burst activities following formalin injection (Fig. 5A1 before and Fig. 5A2 after formalin). The summed burst activities are shown in the histogram in which aligned bursts sequence shows major peaks after the first bust spike (Fig. 5B2) following the formalin injection as compared with the burst histogram in pre-formalin injection period (Fig. 5B1).

The unit activities were recorded *in vivo *in a region of MITN that was confirmed to receive a signal from the foot. The spike pattern was further confirmed to project to the ACC by the spike-triggered averaging technique. This bursting pattern of thalamic discharges can be converted to a irregular bursting stimulation pattern which was then used to evaluate the STP of ACC activity *in vitro*.

### MITN-Pattern Stimulation: Burst and Glutamate Dependence

**Figure 6:**
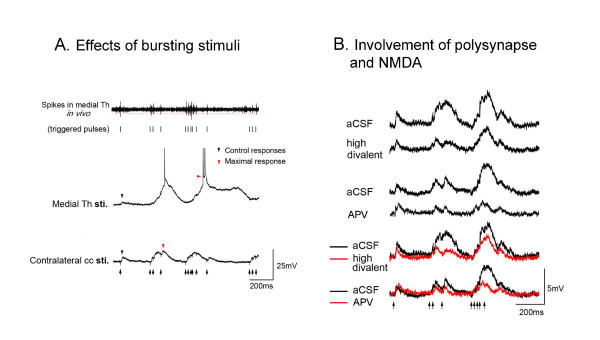
**Differential effects of patterned-bursting stimuli on ACC neurons in MITN-ACC slice**. **A**. EPSPs were recorded from ACC neurons when the in vivo bursting stimulus pattern was applied at the thalamus, or contralateral corpus callosum in the slice. **B**. EPSP potentiation by MT bursting stimulation is shown in the upper sweeps. The EPSPs evoked by the same stimuli during perfusion of high divalent aCSF or APV. The effects are illustrated with superimposed sweeps obtained during control (black lines) and in high divalent and APV conditions (red lines) (Modified from 46).

Figure 4 shows the *in vitro *stimulation/recording paradigm for studying ACC discharges following burst-modelled stimulation patterns that simulate physiological responses. Thalamic bursting unit patterns obtained following formalin injection were first transformed into transistor-transistor logic pulses. These square wave pulses meet the specific requirement for the triggering of an output signal generated from a pulse generator. These triggered stimulating pulse sequences were then used to apply stimulation in the thalamus or contralateral cc and to evoke EPSPs in ACC neurons in slices. The amplitude of EPSPs evoked by the first four stimuli from different stimulation sites were measured and normalized relative to the first responses. The thalamus-evoked fourth EPSPs showed greater potentiation than that produced by contralateral cc (Fig. 6A). The maximal response that occurs during the train of thalamus bursting was measured. Analyses of both the maximal-to-control ratios of the EPSPs and the number of evoked action potentials showed that thalamic stimulation efficiently delivered the bursting unit pattern to the ACC and elicited substantial cingulate neuron firing.

There is evidence that the thalamocingulate projection is glutamatergic [45] and a blocker of the NMDA receptor is d, l-2-amino-5-phosphonopentanoic acid (APV) and it was used to evaluate the role of glutamate in this system. As shown in Figure 6B, the potentiation of the EPSPs by thalamic bursting stimuli was diminished following perfusion with APV (30 μM) in contrast to artificial cerebrospinal fluid (aCSF). Thus, this pathway uses glutamate as is characteristic of all thalamocortical projection systems [69,70].

Another way to analyze the monosynaptic, thalamocortical response in the slice and to dissociate it from multisynaptic, intracingulate activity is to use a high concentration of divalent cations. This protocol was used by Sah and Nicol [66] in cingulate cortex *in vitro *while stimulating the corpus callosum and recording from layer V neurons. Here we use the high divalent cation solution to differentiate the monosynaptic thalamic projection and potentially confounding reverberating cingulate excitatory connections. This protocol also provides a means of diminishing responses to bursting stimuli that generate potentiation by polysynaptic circuitry presumably by increasing the threshold for spike generation in interneurons.

The potentiation induced by thalamic burst stimulation was significantly reduced after high divalent cations in comparison to aCSF. Figure 6B shows that the first response was not affected by either APV or divalent cations, while the 2-5 potentiated responses were highly vulnerable to both treatments. These results indicate that STP of the thalamocingulate pathway provides a specific means by which the MITN can signal to the ACC. Glutamate transmission via NMDA receptors appears to play an important role in transduction of both the initial excitation and subsequent multisynaptic events evoked by nociceptive stimulation. The short-term facilitation observed in the thalamocingulate pathway could enhance the ability of this neuronal circuit to sustain persistent activity evoked by noxious stimulus. Furthermore, such synaptic enhancement could temporarily increase the level of recurrent excitation throughout the local cortical network. Thus the STP may serve to enhance neural activity in cingulate cortex and to adapt this pathway to its specific nociceptive function.

### STP in Layer II/III Involves Pre- and Post-synaptic Mechanisms

Short-term synaptic plasticity shapes the postsynaptic response to bursts of impulses and is crucial for plastic changes of central neurons after strong noxious stimulation. For instance, central sensitization is an enhanced responsiveness of central nociceptive neurons to innocuous and noxious stimuli [27]. All of these plastic changes arise from activity-dependent changes in the amount of neurotransmitter released by persistent actions of calcium ions within the presynaptic terminal [71,72]. The relationship between calcium and STP has been studied in the central nervous system [42,73]. These studies indicated that STP is mediated by the residual calcium in presynaptic terminals which increases the number of quanta released by a second afferent pulse. In light of the fact that calcium acts as an important regulator of PPF [17,74,75], we posited that [Ca^2+^]o is an important regulator of STP in ACC neurons.

We used low frequency, paired-pulse and high-frequency tetanus stimuli to examine STP of the layer II/III neurons in ACC *in vitro *[76]. Also, the post-tetanic effects were tested by altering the time delay between the tetanus and the test stimulus. The effects of different extracellular calcium concentrations and calcium-channel antagonist ω-Conotoxin on STP were examined. For these experiments the stimulation pulses were delivered at different time points at homo-synaptic inputs. To evaluate the effects of stimulating hetero-synaptic inputs in layer II/III, a two-site stimulation method was adopted [77].

**Figure 7:**
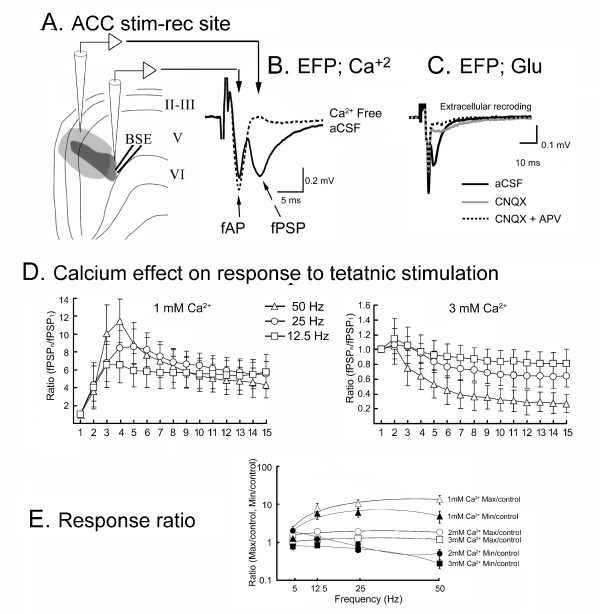
**Responses in the ACC evoked by paired-pulse stimulation**. **A**. The part of ACC in which the half-maximal response for the first field potential (dark gray area) and second field potentials (light gray area) was determined following electrical stimulation in the deep layers (BSE, bipolar stimulating electrodes). **B**. The evoked-field potentials (EFP; either field action potential-fAP or field postsynaptic potential-fPSP) were recorded and fPSPs totally abolished in the presence of Ca^2+^-free aCSF. **C**. Effects of CNQX (15 μM) and APV (30 μM) on EFP. **D**. Frequency-response dependence in the presence of low external [Ca^2+^] (1 mM) aCSF and high [Ca^2+^] (3 mM) aCSF. **E**. Relationship between the ratio of max/control, min/control and frequency under the different external [Ca2+] concentrations (Modified from 66).

The experiments were conducted after finding the location of maximum synaptic response in layer II/III. Two distinct negative potentials were evoked in layer II/III by electrical stimulation applied in layer VI under normal aCSF. Following 200 μA stimulation, the first field potential had a latency of 2.3 ± 0.5 ms and an amplitude of -0.46 ± 0.15 mV and the second field potential had a latency of 6.2 ± 0.7 ms and an amplitude of -0.47 ± 0.095 mV. The evoked potentials were systematically mapped along and around the trajectory path of the layer V/VI neurons. Isopotential plots depicting the areas of the half maximal of the peak amplitudes of the first and second evoked field potentials (EFP) can illustrate the excitatory extent of the evoked responses (Fig. 7A). The maximum potential was obtained in layer II/III for both the first and second field potentials with a slight shift in the distributed location. When the perfusion solution was changed to Ca^2+^-free aCSF, the first field potential was maintained at the same amplitude, but the second field potential was totally abolished (Fig. 7B).

To characterize the cellular components that corresponded to the evoked extracellular field potentials, intracellular excitatory post-synaptic potentials (EPSPs) were evoked and recorded simultaneously with the field potentials. EPSPs and the second field potentials were blocked completely in the presence of CNQX (15 μM) alone or in combination with APV (30 μM). The first field potential however was not affected by the glutamate receptor antagonist (Fig. 7C).

Two consecutive stimuli of identical strength were applied at an interval of 40 ms (paired-pulse stimulation). The field action potential (fAP) evoked by the first stimulus (fAP1) did not differ from that evoked by the second stimulus (fAP2) (amplitude = -0.46 ± 0.15, fAP1 vs. -0.45 ± 0.16 mV, fAP2, stimulated at 200 μA, n = 6). There is a linear relationship between the fAPs and the stimulation strength applied (from 20 to 500 μA). The field post-synaptic potentials (fPSPs) of the second stimulation (fPSP2) had greater amplitude than the fPSPs evoked by first stimulation (fPSP1) (-0.82 ± 0.12 mV vs. -0.47 ± 0.09 mV, stimulated at 200 μA). PPF was obtained with current applied in the range of 20 to 500 μA. Optimal PPF was obtained when the interpulse interval was in the range of 20-150 ms.

These findings suggest that the first fAP was directly activated by stimulation without synaptic relay and the second fPSP resulted from postsynaptic excitation. Strong short-term potentiation of the fPSP of the ACC can be obtained by PPF. Thus, layer II/III neurons express an STP that is calcium dependent and involves glutamatergic transmission. To test the presynaptic mechanism of the PPF, the effects of varying external calcium concentrations and blocking calcium influx were examined.

### Presynaptic Mechanism of STP: Effects of Calcium and ω-Conotoxin

The fPSP response was recorded following 25 Hz (15 pulses with 40 ms IPI) tetanic stimulation under normal calcium conditions with superimposed responses to altered stimuli. The fAP amplitude changed very little suggesting the persistence of the excitability of a pre-synaptic volley. The amplitude of the response following the first stimulus was -0.32 ± 0.017 mV and was regarded as the control value. The fPSP response reached the maximal amplitude (-0.49 ± 0.02 mV, n = 19) following the second stimulus. The maximal response declined gradually in the following stimuli. There was depression of the amplitude after the 10^th ^stimulus and the depressive effect reached a steady state that lasted from the 12^th ^to15^th ^stimuli (-0.223 ± 0.001 mV, n = 19). To evaluate the augmentation effect after tetanus, a single test stimulus was applied at a varying delay interval (0.2~8 s) after cessation of tetanus. The maximal response (-0.48 ± 0.04 mV, n = 19) was obtained when the test stimulus was applied with a 4 s delay.

During tetanic stimulation (12.5 Hz, 25 Hz and 50 Hz), the second fPSP showed maximal facilitation under normal (2 mM) and high (3 mM, Fig. 7D, right plate) calcium conditions. In the presence of low (1 mM, Fig. 7D, left plate) calcium aCSF, maximal fPSP amplitude was reached after the third stimulus presentation and the fPSP gained a much greater Max/control ratio (2~6) than that obtained under normal and high calcium concentrations (1.2~1.8). The fPSP could not be successfully initiated when the extracellular calcium concentration was less than 1 mM. Thus 1 mM was set as the lower limit for the calcium concentration. The fPSP reached a near steady state at the end of tetanus in different calcium concentrations. The Min/control ratio (the ratio of the 15^th ^response to the first response) showed depression in the presence of normal and high calcium aCSF (except at 12.5 Hz stimulation in normal calcium concentration) but showed facilitation in the presence of low calcium aCSF (except at 50 Hz stimulation). The Max/control and Min/control ratios obtained during tetanus under different calcium concentrations were plotted against stimulus frequency as shown in Figure 7E.

The effect of low calcium concentration on fPSP amplitude may be due to a reduction of calcium influx from the extracellular space. To test this possibility, we pharmacologically reduced calcium influx by applying the calcium-channel blocker ω-Conotoxin GVIA (CTX; 10 μM, 3 min in aCSF). The fPSP amplitudes were decreased following CTX application, and the maximal effect was reached within 10 min. We measured CTX effects on facilitation and response depression under tetanus only after stable control responses had been obtained. Slices were perfused in aCSF with 25 μM CTX for 5 min then returned to perfusion with normal or low calcium aCSF. One-way ANOVA indicated a significant effects CTX and low calcium on amplitude of the fPSP_1_, amplitude of the fPSP_2_, maximal amplitude, first response to the test stimuli (4-s 1^st^) and second response to the test stimuli (4-s 2^nd^)(p's < .05), but not on steady-state amplitude or normalized augmentation amplitudes (4-s 1^st^/fPSP_1_) (p's > .05). When comparing PPF ratio, one-way ANOVA indicated a significant effect of Ca^2+ ^influx on PPF (fPSP_2_/fPSP_1_) and PPF during augmentation (fPSP_2 _at 4 s/fPSP_1 _at 4 s).

The results indicated that the presynaptic calcium influx is an important mechanism that regulates the expression of the STP in the layer II/III ACC neurons. As central neurons are susceptible to strong noxious stimulation, the central sensitization of the central neurons may underlie the long-term pathophysiological changes in chronic pain. Thus, the understanding of the calcium regulation in the nociceptive pathway will be crucial in controlling the development of the central sensitization in ACC. A recent study suggested that all different forms of STP may be caused by a common mechanism, namely calcium-dependent regulation of the presynaptic calcium channels that are responsible for triggering transmitter release [72]. It is still to be determined specifically how calcium channel modulation is employed as a mechanism for short-term synaptic plasticity in the thalamocingulate pathway. Recent studies have shown that voltage-gated calcium-permeable ion channels are regulating neuronal excitability, action-potential firing patterns and neurotransmission in nociceptive pathways [78-80]. Thus it will be important in the future to develop new analgesic drugs which target the N-type and T-type calcium channels which are key regulators of nociceptive signaling in humans [81].

### Post-synaptic Mechanisms of STP in ACC

The stimulus protocols applied in all preceding experiments involved consecutive pulses applied to the same synaptic pathway. This paradigm shows the effects of potentiation via single (homosynaptic) inputs. This approach, however, does not allow for the differentiation of STP generated by presynaptic and/or postsynaptic mechanisms. To identify the role of postsynaptic mechanisms, we employ a paradigm in which two separate populations of afferent axons are stimulated independently and the resulting potentiation must be due to the postsynaptic neuron rather than the afferents themselves.

**Figure 8:**
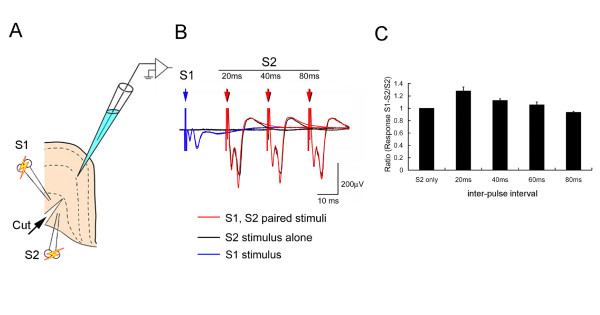
**STP in the ACC evoked by two-site stimulation test**. A. A pre-cut slice was tested with a two-site (S1 and S2) stimulation protocol. B. Averaged and superimposed sweeps generated in response to S2 alone or S1 and S2 paired at varying inter-stimulus intervals (averaged from 5 sweeps). C. Normalized responses evoked by S2 sit stimulation plotted as a function of paired stimulation interval between S1 and S2. Data are expressed as means ± s.e.m (n = 5) (Modified from 66).

A two-site stimulation protocol was used in which two consecutive stimulus pulses were delivered to different axon populations in the subcortical white matter that were separated by a knife cut between the two stimulation sites as shown in Figure 8A. One stimulating electrode is termed the test electrode (S1) and the other is used for conditioning (S2). The evoked-potential amplitude was calculated in ACC that produced half the maximal response evoked by S2. In all sampled recording sites, the majority responses (93%) were facilitated; i.e. the percent change of the fPSP-S2 proceeded with S1 and the fPSP-S2 stimulated alone was greater than 110%. The time course of this effect was also examined, in which facilitation was shown from 20 ms to 60 ms after S1 site stimulation (Fig. 8B). The maximal facilitation effect (ratio = 1.34 ± 0.056) was obtained with a 20 ms interval (Fig. 8C).

Our recent studies have shown that the post-synaptic mechanism of short-term synaptic facilitation in the ACC may be mediated by postsynaptic AMPA and GABA_A _receptors [82,83]. These findings are inconsistent with results obtained in experiments using the lateral amygdala and hippocampal area CA1 [84,85]. Regulation of STP, especially PPF, has been associated with a migration of AMPA receptors [86]. This mechanism would require that AMPA receptors migrate rapidly in the postsynaptic membrane to a position near the glutamate releasing point. In concordance with this hypothesis, Li et al. [87] showed that CNQX binding to AMPA receptors changes their molecular structure and surface charges. Additionally, postsynaptic effects on GABA_A _receptors during PPF may be mediated through a change in the intrinsic membrane excitability triggered by inhibitory post-synaptic, potential-induced hyperpolarization. Hyperpolarization of the neuronal membrane, induced by the GABA_A_inhibitory receptor system, can result in an increase in the second excitatory response during paired-pulse stimulation [77,88].

The facilitation effect observed from multi-site synaptic input was smaller than that from homosynaptic inputs in the same recording area. Our spatial recordings also revealed that not all post-synaptic neurons in the recording area were facilitated by the two-site stimulation protocol. Thus, homosynaptic facilitation may play a major role in modulating information from the same synaptic inputs. On the other hand, multi-site synaptic facilitation may play a role in the convergence of information from different synaptic inputs. Multi-site synaptic interactions, such as depression and facilitation, may differ depending upon the inputs stimulated and the target neurons affected. Post-synaptic facilitation driven by multi-site synaptic inputs may represent an additional signal processing mechanism in the ACC. Both homosynaptic and multi-site synaptic facilitation effectively transfer signals and each has distinct facilitation properties which may be used to distinguish signals received from different origins.

### Implications of Short-Term Plasticities to Nociception in the Thalamocingulate Pathway

Short-term modifications in cortical synapses appear to regulate afferent signals and this regulation is likely important to the transition from acute nociceptive stimulation to chronic pain conditions associated with persistent peripheral noxious stimulation. Our studies have shown that the input-specific, short-term synaptic plasticity in the ACC can enhance signals originating in the MITN. Thus MITN discharges within a certain frequency range are amplified and effectively stimulate cingulate cortical neurons. The thalamic drive of ACC is not a stable event, but rather changes over time depending upon the nociceptive history of the organism. Our studies have shown that cingulate circuits, in addition to being facilitated by regular and repetitive impulses, can be activated by irregular bursting thalamic impulses and that the later impulses within each burst are more likely to elicit action potentials for further signal propagation.

**Figure 9:**
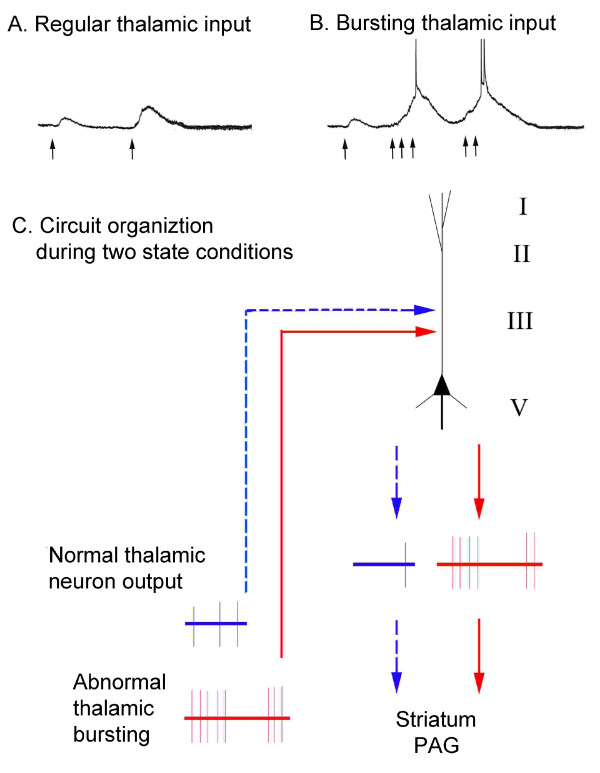
**Diagrams of nociceptive inputs and STP in the ACC under acute normal and abnormal chronic pain conditions**. A. Synaptic responses of ACC neurons to regular discharging thalamic afferents. B. Responses of ACC neurons to bursting thalamic afferents. C. ACC circuit organization during two conditions of thalamic output (cortical layers shown on right). In the acute pain state, the nociceptive signals are conveyed to the ACC through normal thalamic activity to dendritic targets of pyramidal neurons in layer V (blue dashed lines) and evoke a weak discharge. STP in these synapses regulates ACC neuron output to the striatum and PAG. In contrast, abnormal thalamic bursting occurs following persistent nociceptive inputs from the periphery (solid red lines) and these bursting impulses initiate and facilitate multiple action potentials in ACC neurons. The enhanced and maintained activities in these neurons facilitate the intracortical nociceptive transmission and descending outputs which further influence ACC targets in other brain regions.

The potential role of STP in the ACC in nociceptive signaling involved in acute and chronic pain states is presented in a diagram in Figure 9. The synaptic response (e.g. EPSP) of ACC neurons to thalamic input is enhanced by spikes with regular frequency (Fig. 9A.). These enhanced synaptic responses can also be measured as potentiated extracellular field potentials or localized sink currents. Synaptic responses are further potentiated by irregular thalamic bursting, resulting in multiple action potentials (Fig. 9B.). STP of ACC pyramidal neurons in layer V regulates nociceptive signaling relayed from the MITN under normal conditions. Facilitated synaptic responses may lead to a few action potentials in the neuron (blue dashed lines Fig. 9C). These cingulate cortical output signals may be greatly enhanced following the activation of abnormal thalamic bursting in chronic pain conditions and thus influence subcortical targets such as the periaqueductal grey (PAG) and striatum (Fig. 9C., solid red lines).

Considerable effort has been made to understand the mechanisms underlying high-frequency bursting of thalamocortical impulses. It has been shown, for example, that initial impulses of each burst have a greatly enhanced ability to elicit cortical action potentials, and later impulses in the burst further raise the probability of eliciting spikes [89]. Moreover, the interval preceding each burst is crucial for generating the enhanced cortical response. The properties of thalamic burst mode have led to suggestions that bursts could serve as wake-up calls to cortex for potentially dangerous stimuli. The bursting activity has been reported in the medial thalamus of rats in normal condition and with chronic inflammation [90]. If STP in ACC regulates the acute nociceptive input signals from the thalamus, then this property may play an important role when abnormal thalamic bursting occurs in pathological pain conditions. Thus, the potentiation induced by bursting patterns of stimulation indicate that short-term synaptic plasticity in the cingulate neurons enable them to process specifically the nociceptive information relayed from MITN.

Several groups have reported the existence of thalamic neurons in chronic pain patients that fired in a bursting pattern similar to the low-threshold calcium spike-mediated bursting activity and such firing may be the result of and/or cause of chronic pain. Recent results indicate that T-type Ca^+2 ^channels are responsible for burst spike discharges in response to visceral pain and support the idea that burst firing plays a critical role in sensory gating in the thalamus [91]. Unit studies by Rinaldi and co-workers [92] in patients with deafferentation pain found thalamic cells with high frequencies of spontaneous bursting discharge activity. The receptive fields of these units were very large and often bilateral. An increase in the relative rates of spontaneous activity in the thalamus has been reported for central pain patients as compared to non-pain patients [93]. A study by Jeanmonod and coworkers [94] shows that 50% of thalamic units in chronic neurogenic pain patients showed random and rhythmic bursting activities. The rhythmic bursting units were characterized by interburst intervals between 200 and 300 ms. Random bursting units showed a more or less marked rhythmic tendency toward these frequencies. The first spike of each burst was often of higher amplitude, and interspike intervals within a burst increased with each successive interval (from 2 to 8 ms). The shorter the first interspike interval in a burst, the larger the number (4 to 10) of spike was found within this burst. All of these characteristics are the hallmark of low-threshold, calcium spikes (LTS) bursts [95]. Much of the increase in these activities in these reports may be accounted for by increased spontaneous bursting activities of medial thalamic cells. For example, the bursting activity patterns found by Rinaldi et al. [92] was concentrated to the lateral aspect of the mediodorsal nucleus, the central lateral nucleus and only a small part of the central medial-parafascicularis complex. The anatomical distribution of the bursting units in Jeanmonod's study shows a clustering in and around central lateral and ventrocaudal part of the mediodorsal nucleus.

The significance of the bursting pattern found by these authors has been interpreted to occur by a mechanism attributed to intra-thalamic interactions. Jeanmonond et al. proposed that the spinal inputs to medial and lateral thalamus are excitatory, and that the excitation of each region is limited by inputs of each area to the reticular thalamic nucleus which then produces a reciprocal inhibition of the medial thalamus by the lateral thalamus and vice versa. They suggested that most injuries resulting in chronic pain tend to deprive the lateral more than medial thalamus of peripheral inputs. Thus, the lateral thalamus becomes over inhibited by a combination of loss of spinal excitatory inputs and increase of inhibitory inputs from the thalamic reticular nucleus. These combined influences produce over-inhibition of lateral thalamic cells producing low-threshold calcium spikes. This spiking activity then over activates the reticular thalamic projection back to medial thalamus which finally produces low-threshold calcium spiking in this region and so closes a self sustaining loop of over activity through inhibition. In this model calcium spike associated bursting requires the combination of excitatory amino acid antagonists with GABA agonists.

Although the medial thalamic LTS bursting activity emphasizes the thalamic mechanism of chronic pain, the potential pathophysiological relevance of thalamo-cortico-thalamic reverberating and/or synchronizing loops should not be underestimated. The medial thalamus was shown to be preponderant in the genesis of rhythmic thalamic oscillations [96]. The widespread cortical projections of the medial thalamic nuclei have been shown to influence the activity of a large number of cortical areas when functioning in bursting mode [97]. Thus, the specific short-term facilitation of the thalamocingulate pathway will likely enable the enhancement of the transferring of the abnormal thalamic bursting activities to the cingulate cortex. These activities will result in a resonant interaction between thalamus and cingulate cortex and thus sustained nociceptive activities.

The STP time scale is relatively short, on the order of seconds and minutes, and thus it cannot produce entirely the processes underlying chronic pain conditions. It is crucial to note that STP plays a transitional role in transferring the nociceptive signal mediating acute traumatic injury to the formation of long lasting changes in the ACC. Long-term enhancement of synaptic transmission after peripheral injury has been demonstrated in several studies in which the potentiation of the peripherally evoked field and EPSP in the ACC lasted 90 ~120 min or longer. [98,99]. These long-term effects were further validated in experiments in genetically modified mice showing that immediate early genes were activated in ACC neurons after peripheral inflammation or amputation [100,101]. The expression of the immediate early gene expression involved activation of NMDA receptors and two subtypes of adenylyl cyclase (AC1 and AC8) [101,102]. The molecular mechanism underlying the long- term plastic changes in the ACC were found to be related to the NMDA receptors and L-type voltage-gated calcium channels, which are responsible for the induction of the long-term potentiation (LTP) of ACC synaptic responses [103,104]. NMDA receptors without Ca^2+ ^permeable GluR2 subunits were found to be critical to LTP stabilization and maintenance [105].

The short-term and long-term plastic changes in the ACC may facilitate the acute nociceptive activities to become persistent. Enhanced and maintained nociceptive activities may have an adverse effect in cingulate cortex. Studies have shown that excessive activation of NMDA receptors which are the most widely and densely distributed of the glutamate receptor subtypes in the cingulate cortex, plays an important role in the pathophysiology of acute CNS injury syndromes [106]. NMDA antagonists are being used or evaluated for use in chronic conditions like neuropathic pain [107]. Although there are clinical reports suggesting a role of NMDA-antagonists in chronic neuropathic pain [108,109], the exact clinical role of NMDA-blockade remains to be investigated. Experimental evidence points at a substantial role of the NMDA-receptor initiating central sensitization that possibly lead to persistent pain-states [110,111]. Therefore, the use of NMDA-receptor antagonists in the early post-injury phase, may pre-empt central sensitization and the subsequent development of chronic pain. From this point of view, the therapeutic efficacy of NMDA-antagonists may not act on central sensitization once chronic pain is established. It is possible that it may instead act in the regulation of the excessive cortical excitation of NMDA receptors involved in other pathophysiological events.

Thalamic T-type calcium channels are critically involved in the generation of burst firing and oscillatory behavior in synaptically interconnected relay and reticular neurons. Of particular note, CaV3.1 channels in the thalamus have been implicated in processing of noxious stimuli [91]. The presence of a thalamocortical dysrhythmia is due to the generation of low-threshold, calcium-spike bursts by thalamic cells. The presence of these bursts is directly related to thalamic cell hyperpolarization, brought about by either excess inhibition or disfacilitation. Thus, it will be particularly important to know how to regulate such changes in thalamic firing patterns that may influence the transmittal of nociceptive information to the ACC. One approach of the thalamic firing modification may be the application of calcium channel blockers. For instance, it is possible that selective targeting of CaV3.3-expressing neurons in the reticular nucleus could inhibit γ-aminobutyric acid release. In turn, this would lead to less hyperpolarization of neurons in the relay nuclei with decreased availability of their T-type calcium channels. The consequence could be a switch from phasic to tonic firing with interruption of pathological rhythmic and oscillatory electrical activity.

## Conclusions and Unresolved issues

Short-term synaptic plasticities play an important role in the processing of input signals by enhancing or filtering signals at particular frequencies. In the ACC, unlike the primary sensory cortical areas, paired-pulse facilitation is predominant. The STP features, including homo-, hetero-synaptic facilitation and augmentation, keep neuronal activity propagation within local circuits. Thus, the potentiation induced by bursting patterns of stimulation indicate that short-term synaptic plasticity in the cingulate neurons enable them to process specifically the nociceptive information relayed from the MITN. Short-term modifications in cingulate cortical synapses appear to regulate afferent signals and this is likely very important to the transition from acute nociceptive stimulation to chronic pain conditions associated with persistent peripheral noxious stimulation.

Enhanced and maintained nociceptive activities in cingulate cortex may have an adverse effect. Thus, it has been suggested to use NMDA antagonists in chronic conditions like neuropathic pain. Thalamic, T-type calcium channels are critically involved in the generation of burst firing and oscillatory behavior in thalamocortical dysrhythmia. Thus, it will be particularly important to know how to regulate such changes in thalamic firing patterns that may influence the transmittal of nociceptive information to the ACC. One approach of the thalamic firing modification may be the application of calcium channel blockers.

## Competing interests

The authors declare that they have no competing interests.

## Authors' contributions

BAV initiated the idea and BCS drafted the manuscript. BAV revised it critically for important intellectual content; both authors read and approved the final manuscript.
